# Equity, diversity, and inclusion topics at a medical physics residency journal club

**DOI:** 10.1002/acm2.14126

**Published:** 2023-08-15

**Authors:** Jessica M. Fagerstrom, Cheyann Windsor, Daniel Zaks

**Affiliations:** ^1^ Department of Radiation Oncology University of Washington Seattle Washington USA; ^2^ Northwest Medical Physics Center Lynnwood Washington USA

**Keywords:** diversity, education, equity, inclusion, journal club, medical physics, residency

## Abstract

A journal club program was initiated in a clinically focused, geographically distributed medical physics therapy residency program. This program currently supports two residents at different clinical sites, who regularly present at the new journal club. For one of the sessions, residents were assigned to present on topics related to the broad themes of equity, diversity, and inclusion (EDI) in the context of medical physics, radiation oncology, or medical oncology. As in other journal club sessions, residents were responsible for choosing their respective articles within required criteria and with approval from the program director. The session was executed in late 2022, with both residents leading and facilitating discussion for the residents, the residency program director, and all residency faculty members. This education case report will include the learning objectives for the journal club session, a description of the content covered in the session, discussion regarding the session's alignment with the original learning objectives, and ideas for program directors intending to include evidence‐based EDI topics in journal clubs.

## 1 INTRODUCTION AND NARRATIVE

As discussed by professional organizations including the American Association of Physicists in Medicine (AAPM), American Board of Radiology (ABR), Accreditation Council for Graduate Medical Education (ACGME), and American SocieTy for Radiation Oncology (ASTRO), equity, diversity, and inclusion (EDI) are important topics in professional medical education.^1^, ^2^, ^3^, ^4^ For this work, the definitions for these broad concepts are as noted by the American Medical Association (AMA) and Association of American Medical Colleges (AAMC).^5^ Here, equity “refers to fairness and justice and is distinguished from equality. While equality means providing the same to all, equity requires recognizing that we do not all start from the same place because power is unevenly distributed.” Diversity “refers to the identities we carry. There are many kinds of diversity, based on race, gender, sexual orientation, class, age, country of origin, education, religion, geography, physical or cognitive abilities, or other characteristics. Valuing diversity means recognizing differences between people, acknowledging that these differences are a valued asset, and striving for diverse representation as a critical step towards equity.” Inclusion “refers to how our defining identities are accepted in the circles that we navigate. […] Inclusion is a state of being valued, respected and supported.” While other organizations may have differing definitions for the concepts defining equity, diversity, and inclusion, the AMA and AAMC definitions were deemed appropriate for the purposes of the journal club session and are adopted within this work for common understanding.

EDI is an active area of research within the field of medical physics. The Commission on Accreditation of Medical Physics Education Programs (CAMPEP) Board is currently drafting standards addressing EDI that will apply to all CAMPEP‐accredited educational programs, including graduate, doctorate of medical physics, certificate, and residency programs (email communication, 6 August, 2022), and the update to AAPM's TG‐249 regarding guidelines for clinical medical physics residency programs, currently under review, contains a section on EDI (email communication, 8 May, 2023). Based on the critical need to increase equitable opportunities and workforce diversity and inclusion within the medical physics profession,^6^ a session of a CAMPEP‐accredited medical physics residency journal club was dedicated to examining scholarship based on EDI and applying it to current practice.

### 1.1 General journal club format and curriculum

Northwest Medical Physics Center (NMPC) is a non‐profit medical physics service group providing medical physics consulting services to a variety of clinics throughout a geographically distributed region. They have sustained a CAMPEP‐accredited residency program in therapeutic medical physics since 2009, and currently support two medical physics residents who are stationed at two different clinical sites. The program hosts a quarterly, resident‐led journal club, modeled after best practices described by Cetnar.^7^ Residents are responsible for choosing their own article(s) to present, with the aim to promote learning how to review available literature and to encourage residents to build the skills surrounding searching different resources effectively. Chosen articles must be peer‐reviewed; related to medical physics, radiation oncology, or medical oncology; and centered on evidence‐based medicine (scientific or professional topics), but there is broad latitude in article choice. All presentation topics must be approved by the faculty journal club leader.

During the videoconference journal club meeting, the first presenting resident gives a prepared presentation, approximately 20 min long, that includes an introduction to the article(s), a discussion of why the selection was made, a review of key components, a critical analysis, and suggestions on how this work may be applicable to their practice. Following the formal presentation, the non‐presenting resident discusses how this work may or may not be applied to their own clinical site or to procedures they have seen in the past. After both residents have discussed the article(s), the presenting resident then leads and facilitates a discussion with all journal club members, discussing clinical and/or professional application of evidence‐based scholarship. This facilitated discussion lasts approximately 20 min. Faculty members are encouraged to contribute, but they are instructed not to dominate the discussion. All contributors are required to engage in respectful discourse. After the first resident has presented and facilitated a discussion for their topic, the second resident presents and facilitates the second topic. Feedback from all journal club members is collected following each journal club session, with possible adjustments made to following sessions based on this feedback.

**LEARNING OBJECTIVES**

The following learning objectives were proposed for a journal club session focused on topics of equity, diversity, and inclusion at a medical physics residency program.

- Residents will improve in professional scientific presentation skills and critical thinking/analysis of scientific, peer‐reviewed literature.
- Journal club members will better understand challenges faced by people of different identities, including those related to race, ethnicity, culture, gender, age, sexual orientation, ability, religion, economic status, language, national origin, and other backgrounds within the context of medical oncology, radiation oncology, and medical physics.
- The equity, diversity, and inclusion (EDI) journal club session will promote discussion about applying EDI topics in professional settings among club members.

### 1.2 Theoretical framework

A theoretical framework is the lens through which scholarly work is approached, including the underlying concepts and theories that inform scholarly work.^8^ The theoretical framework for this educational case study report is informed by social constructivism,^9^ which emphasizes the collaborative characteristics of knowledge construction, and highlights that an individual student's learning develops within the context of their broader environment, including cultural and other social contexts.^10^ Within the broad category of social constructivism, this journal club used the more specific theoretical framework of communities of practice. Communities of practice involve diverse groups of people who share abilities, delve into common interests and issues, and collectively develop knowledge.^11^ This framework has been applied to education scholarship in many contexts, including medical and health care education,^12^ and is the framework for this journal club.

### 1.3 Innovative elements

Journal clubs are a conventional element of medical education, and these journal clubs frequently consist of evaluating and critiquing scientific articles revolving around evidence‐based medicine.^13^ Journal clubs within medical physics residencies, including at the current institution, tend to focus on clinical, technical, and scientific research.^7^ To the best of the authors’ knowledge, there are currently no published accounts of EDI‐based journal clubs in medical physics, radiation oncology, or medical oncology. A departure from this standard with a session devoted to EDI topics (traditionally underrepresented topics within medical physics journal clubs) can showcase that EDI topics can be studied and critiqued in a rigorous way. These types of sessions introduce both faculty and trainees to the wide and growing array of available peer‐reviewed literature. Further, this session offers a unique learning opportunity allowing individuals from a variety of backgrounds to share professional experiences related to EDI within a community of practice of clinical physicists, with the possibility of fostering a more EDI‐focused workforce.

### 1.4 EDI journal club session

For the EDI journal club session, residents were required to focus on studies related to EDI in the context of medical physics, radiation oncology, or medical oncology. The journal club leader selected eight peer‐reviewed articles that they believed could inspire thoughtful EDI discussion as example options. Residents were invited to pick from the list or make their own choices, with the request that any choices outside the list be discussed with the program director prior to finalizing the article choice. Each resident was required to present two articles. Residents were instructed to facilitate discussion regarding practical applications from each paper in relation to themes of EDI: what NMPC as a residency and the journal club members as medical physics colleagues can learn and improve on based on this work. The pre‐selected paper collection is included below:

- “Diversity based on race, ethnicity, and sex, of the US radiation oncology physician workforce,” by Chapman, Hwang, and Deville^14^
- “Ethical violations and discriminatory behavior in the MedPhys Match,” by Hendrickson et al.^15^
- “The state of gender diversity in medical physics,” by Covington, Moran, and Paradis^16^
- “I can't breathe: The continued disproportionate exclusion of black physicians in the United States radiation oncology workforce,” by Deville et al.^17^
- “Implications of medical board certification practices on family planning and professional trajectory for early career female radiation oncologists,” by Dover et al.^18^
- “A qualitative investigation of resilience and well‐being among medical physics residents,” by Paradis et al.^19^
- “Diversity and professional advancement in medical physics,” by Rankin et al.^6^
- “2021 AAPM Equity, Diversity, and Inclusion Climate Survey Executive Summary,” by Hendrickson et al.^20^

The EDI‐themed journal club was presented in late 2022. The first presenting resident led discussion on one article included in the list above, Hendrickson et al.^15^ and one article chosen independently from the list, based on resident interest and direct applicability to the resident's practice: “Disparities in access to radiation therapy facilities among American Indians/Alaska natives and Hispanics in Washington State” by Greer et al.^21^ The second presenting resident led discussion on two articles included in the list above, Paradis et al.^19^ and Rankin et al.^6^ In addition to the two resident leaders, all five faculty members (including the program director) attended.

## 2 DISCUSSION

At the journal club session, the residents led a nuanced discussion about major EDI issues, including biased and discriminatory environments faced by some trainees, culturally competent care offered to patients, and disparate access to care for some patient populations. For the discussion regarding the article focused on ethical violations and discriminatory behavior in the MedPhys Match, the resident described their own personal experience with some of the illegal and unethical questions raised during their own match interview process. They asked the group to think about how questions regarding marital status or children may affect candidates in varying personal situations, such as candidates planning to marry their partner, candidates going through a divorce, or candidates experiencing pregnancy. For the discussion regarding the article evaluating disparities in access to radiation therapy among American Indian/Alaska natives and Hispanic patients in Washington State, the resident discussed resources and funding programs at their clinical site of practice to make care more accessible to patients with different levels of resources. Some of the examples discussed were parking available for RV trailers near the radiotherapy center, and social workers being actively involved in patient care, including attending and contributing to radiation therapy daily huddle mini‐conferences. A major discussion question the resident posed based on this article was regarding what medical physicists can do to better support accessibility efforts in their own communities. For the discussion regarding the article focused on diversity and professional advancement in medical physics, the resident posed questions to the journal club regarding how childcare needs and inequitable leadership promotion practices negatively impact some groups of physicists compared to others. The resident reviewed the lack of discussion within the paper regarding experiences of marginalized groups, but also noted that the paper included some suggestions on combating a lack of diversity that may be valuable. Among other topics, the resident asked the journal club members to consider ways to improve mentorship and networking opportunities for medical physicists and medical physics residents, as well as provide social, financial, and institutional support. For the discussion regarding the final article examining resilience among medical physics residents, the resident posed questions about the role of professional societies in addressing residency‐related change, including focusing on widespread burnout, strategies to cope with residency‐related stress, and the demanding nature of the match process, especially for candidates who may face additional barriers or discrimination.

These topics are of importance to the AAPM, which adopted diversity and inclusion as components of their focus areas in 2018, with the strategic goal of championing equity, diversity, and inclusion in the field of medical physics.^1^ Residents and faculty agreed that the journal club session aligned with the learning objectives, with residents providing well‐researched presentations and fostering an environment conducive to learning for all journal club members. Following the EDI journal club session, residents were asked to evaluate statements using a 5‐point Likert scaling, with 1 indicating they strongly disagree, 2 that they disagree, 3 that they neither agree nor disagree, 4 that they agree, and 5 that they strongly agree. The mean scores for the following statements “I found the EDI journal club session personally valuable,” “I had the opportunity share my views on my chosen articles,” “Discussing EDI topics can be useful in the context of medical physics residency,” and “I felt I had sufficient time to examine my chosen articles to the extent I wanted” were 4.5, 5.0, 5.0, and 4.5, respectively.

Educators interested in leading sessions dedicated to EDI topics in medical physics journal clubs, including those involving residents, are encouraged to review available resources, including emerging scholarship. Note that journal club leaders should review the body of literature beyond the list included earlier, as this area of research is currently active, and the available literature is continually expanding. For example, since the list included in the previous section was compiled, there have been thought‐provoking, evidence‐based articles, reviews, and book chapters published on ethics and artificial intelligence in radiation oncology,^22^ EDI in radiation oncology residency programs,^23^ global inequities in radiation therapy access in low‐ and middle‐income countries,^24^ inequities in radiation therapy access within the United States,^25^ EDI topics in radiation oncology,^26^ and an EDI climate survey in medical physics.^27^

It is noted that the broad range of publications that could be used to explore EDI topics cover an extensive scope of problems, including the needs to improve workforce diversity, to reduce health care disparities and increase access to quality health care across the patient population, and to foster a culture of belonging within the profession. As noted by Ponce et al.,^25^ these are all important—yet unique—subjects. Journal club leaders should not expect a single journal club session to address all of these topics thoroughly, but instead, journal clubs may elect to hold multiple sessions over time devoted to themes in EDI.

### 2.1 Additional journal club resources

The AAPM currently offers resources for residency educators hoping to host a journal club. The AAPM Work Group on Multi‐Institutional Journal Clubs for Residency Programs (WGJCRP) maintains group guidelines available by request for residency programs that intend to contribute to a distributed journal club (email communication, 4 May 2023). Interest in multi‐institutional journal clubs has been high among CAMPEP‐accredited medical physics residency programs, and the WGJCRP has facilitated the meeting of multiple national medical physics journal clubs since its formation in 2021. As of 2022, 40% of therapy and 50% of imaging and nuclear medicine accredited programs were participating in this voluntary multi‐institutional journal club program through WGJCRP.^28^ Further, the AAPM Professional Council introduced a major leadership initiative, the Medical Physics Leadership Academy (MPLA), in 2016.^29^ Among other activities, MPLA hosts a monthly journal club via videoconference (recently retitled the “MPLA Leadership Club”) with each monthly session focused on a different topic involving leadership within medical physics. Participants review resources such as podcasts, news articles, and other creative resources prior to meeting, and then spend much of the time in small breakout sessions discussing resources and reviewing discussion questions. MPLA previously hosted a “Respect for Diversity” session in 2022,^30^ with thought‐provoking resources including a Harvard Business Review essay and a TEDx talk. The MPLA Leadership Club diversity session and the EDI journal club session described in this work offer complementary approaches to broaching complex EDI topics: one using valuable popular media resources and one relying on research‐based, peer‐reviewed articles. Both methods can result in constructive educational experiences surrounding EDI topics.

## 3 CONCLUSION

A medical physics residency journal club session was held focusing on EDI topics in the context of medical physics, radiation oncology, and medical oncology. Four recent, peer‐reviewed journal articles were presented, with discussion led by residents. Residents and faculty members agreed that the broad objectives of the journal club were met in this session, and resident and faculty members considered the session useful as a platform to discuss EDI. Educators who are interested in incorporating EDI topics into journal clubs are encouraged to review some of the resources discussed in this case report, and acknowledge that all major concepts in equity, diversity, and inclusion cannot be adequately covered in a single journal club meeting. Exploring topics in EDI within the context of a residency journal club offers a complementary perspective on the discipline of medical physics to the usually technical and scientific topics explored in journal club sessions. Highlighting these issues through a journal club helps residents and faculty increase awareness of EDI issues.

## AUTHOR CONTRIBUTIONS

The first author designed and initiated the residency journal club, acted as journal club leader and organizer, researched the theoretical framework for the project, conceived the EDI journal club session, requested and received Steering Committee approval for the session, compiled the literature for the EDI session, coordinated faculty and resident attendance for the session, and wrote the manuscript. The second and third authors had equal contributions to the manuscript. Each researched and reviewed available literature, presented two EDI‐focused articles, and led discussion among the resident and faculty group. They reviewed and revised the manuscript.

## CONFLICT OF INTEREST STATEMENT

There are no relevant conflicts of interest to disclose.

## References

[1] AAPM strategic plan . American Association of Physicists in Medicine website. Published 2018. Accessed May 9, 2023. https://w3.aapm.org/org/objectives.php

[2] New committee works to instill DEI initiatives across ABR. American Board of Radiology website. Published 2022. Accessed May 9, 2023. https://www.theabr.org/beam/from‐the‐board‐of‐trustees‐december‐2022

[3] ACGME equity matters . Accreditation Council for Graduate Medical Education website. Published 2021. Accessed May 9, 2023. https://www.acgme.org/what‐we‐do/diversity‐equity‐and‐inclusion/ACGME‐Equity‐Matters/

[4] ASTRO's commitment to diversity, equity, and inclusion. American Society for Radiation Oncology website. Published 2022. Accessed May 9, 2023. https://mydigitalpublication.com/publication/?i=751596&article_id=4297507&view=articleBrowser

[5] Advancing health equity: a guide to language, narrative and concepts. American Medical Association and Association of American Medical Colleges website. Published 2021. Accessed March 10, 2023. https://www.ama‐assn.org/system/files/ama‐aamc‐equity‐guide.pdf

[6] Rankin J , Whelan B , Pollard‐Larkin J , et al. Diversity and professional advancement in medical physics. Adv Radiat Oncol. 2023;8(1):101057. 10.1016/j.adro.2022.101057 36213550 PMC9539787

[7] Cetnar AJ . Model for implementation of a modern journal club in medical physics residency programs. J Appl Clin Med Phys. 2021;22(6):253‐261. 10.1002/acm2.13250 33987945 PMC8200434

[8] Merriam SB , Tisdell EJ . Qualitative Research: A Guide to Design and Implementation. Jossey‐Bass; 2016.

[9] Vygotsky L . Interaction between learning and development. In: Cole M , John‐Steiner V , Scribner S , Souberman E , eds. Mind and Society: The Development of Higher Psychological Processes. Harvard University Press; 1978:79‐91.

[10] Kalina C , Powell KC . Cognitive and social constructivism: developing tools for an effective classroom. Education. 2009;130(2):241‐250.

[11] Wenger E . Communities of Practice: Learning, Meaning, and Identity. Cambridge University Press; 1998.

[12] le May A . Chapter 1: introducing communities of practice. In: le May A , ed. Communities of Practice in Health and Social Care. Wiley‐Blackwell Pub; 2009:3‐16.

[13] Ilic D , de Voogt A , Oldroyd J . The use of journal clubs to teach evidence‐based medicine to health professionals: a systematic review and meta‐analysis. J Evid Based Med. 2020;13(1):42‐56. 10.1111/jebm.12370 31951092

[14] Chapman CH , Hwang W‐T , Deville C . Diversity based on race, ethnicity, and sex, of the US radiation oncology physician workforce. Int J Radiat Oncol Biol Phys. 2013;85(4):912‐918. 10.1016/j.ijrobp.2012.08.020 23122983

[15] Hendrickson KR , Juang T , Rodrigues A , Burmeister JW . Ethical violations and discriminatory behavior in the MedPhys Match. J Appl Clin Med Phys. 2017;18(5):336‐350. 10.1002/acm2.12135 28834035 PMC5874901

[16] Covington EL , Moran JM , Paradis KC . The state of gender diversity in medical physics. Med Phys. 2020;47(4):2038‐2043. 10.1002/mp.14035 31970801 PMC7217161

[17] Deville C , Cruickshank I , Chapman CH , et al. I can't breathe: the continued disproportionate exclusion of black physicians in the United States Radiation Oncology Workforce. Int J Radiat Oncol Biol Phys. 2020;108(4):856‐863. 10.1016/j.ijrobp.2020.07.015 32668279 PMC7354371

[18] Dover LL , Hentz C , Kahn JM , et al. Implications of medical board certification practices on family planning and professional trajectory for early career female radiation oncologists. Pract Radiat Oncol. 2022;12(2):95‐102. 10.1016/j.prro.2021.08.014 35000892

[19] Paradis KC , Ryan KA , Schmid S , et al. A qualitative investigation of resilience and well‐being among medical physics residents. J Appl Clin Med Phys. 2022;23(3). 10.1002/acm2.13554 PMC8906227 35128786

[20] Hendrickson KRG , Avery SM , Castillo R , et al. 2021 AAPM equity, diversity, and inclusion climate survey executive summary. Int J Radiat Oncol Biol Phys. 2022. 10.1016/j.ijrobp.2022.02.030 35235854

[21] Greer MD , Amiri S , Denney JT , Amram O , Halasz LM , Buchwald D . Disparities in access to radiation therapy facilities among American Indians/Alaska Natives and Hispanics in Washington State. Int J Radiat Oncol Biol Phys. 2022;112(2):285‐293. 10.1016/j.ijrobp.2021.08.038 34715256

[22] Hyun M , Hyun A . Chapter 15: ethics and artificial intelligence in radiation oncology. In: Mun SK , Dieterich S , eds. Artificial Intelligence in Radiation Oncology. World Scientific Publishing Co Pte Ltd; 2023:337‐358.

[23] Williams VM , Franco I , Tye KE , et al. Radiation oncology residency training program integration of diversity, equity, and inclusion: an Association of Residents in Radiation Oncology Equity and Inclusion Subcommittee (ARRO EISC) inaugural program director survey. Int J Radiat Oncol Biol Phys. 2023. 10.1016/j.ijrobp.2023.02.025. Journal pre‐proof. 36828169

[24] Christ SM , Willmann J . Measuring global inequity in radiation therapy: resource deficits in low‐ and middle‐income countries without radiation therapy facilities. Adv Radiat Oncol. 2023;8(4):101175. 10.1016/j.adro.2023.101175 37008253 PMC10050474

[25] LaVigne AW , DeWeese TL , Wright JL , Deville C , Yegnasubramanian S , Alcorn SR . Radiotherapy deserts: impact of race, poverty and the rural‐urban continuum on density of providers and utilization of radiotherapy in the United States. Int J Radiat Oncol Biol Phys. 2023;116(1):17‐27. 10.1016/j.ijrobp.2023.01.046 36736631

[26] Franco P , De Felice F , Kaidar‐Person O , et al. Equity, diversity, and inclusion in radiation oncology: a bibliometric analysis and critical review. Int J Radiat Oncol Biol Phys. 2023. 10.1016/j.ijrobp.2023.02.026. Journal pre‐proof. 36841344

[27] Aldosary G , Koo M , Barta R , et al. A first look at equity, diversity, and inclusion of Canadian medical physicists: results from the 2021 COMP EDI climate survey. Int J Radiat Oncol Biol Phys. 2023. 10.1016/j.ijrobp.2023.01.044. Journal pre‐proof. 36724859

[28] Prisciandaro J , Cetnar A , Lincoln H , et al. A year in review: A summary of Education Council's 2022 activities. American Association of Physicists in Medicine website. Published 2022. Accessed May 9, 2023. https://issuu.com/aapmdocs/docs/4706

[29] Kapadia A , Cetnar A , Sansourekidou P , Wang D , et al. MPLA: The medical physicist's secret weapon for career advancement. American Association of Physicists in Medicine website. Published 2020. Accessed May 9, 2023. https://issuu.com/aapmdocs/docs/4506?mode=embed&viewMode=doublePage&backgroundColor=eeeeee&pageNumber=33

[30] Medical Physics Leadership Academy: Respect for Diversity journal club session . American Association of Physicists in Medicine. Published 2022. Accessed May 9, 2023. https://w3.aapm.org/leadership/community/journalClub/diversity.php

